# Fast Neutron Dose Measurements for a D–D Neutron Source in Water*

**DOI:** 10.6028/jres.068A.001

**Published:** 1964-02-01

**Authors:** W. B. Beverly, V. Spiegel

## Abstract

The penetration of D–D neutrons in water has been studied through measurements of first collision dose. A relative measurement of first collision dose as a function of distance from the neutron source was made at 0°. At large penetrations the results appear to approach asymptotically the slope predicted by the theoretical calculations of Goldstein et al. [0] for a monoenergetic, isotropic 4.0-Mev neutron source in water. However as expected, the measurements close to the source where the average neutron spectrum is relatively soft indicate a steeper slope than the theoretical calculation. Further calculations will be required to obtain an explicit check of this experiment.

## 1. Introduction

This experiment is a continuation of a series of experiments performed at this laboratory pertaining to the slowing down of neutrons in hydrogenous media [1–3].^1^ The 14-Mev experiment in water of Caswell et al. [1] was the only previous one in which an attempt was made to measure fast neutron dose. Fast neutron dose was also measured by Otis [4] using the fission neutrons from a U^235^ disk source. Experiments such as these are needed to check the calculation methods and input data used for neutron shielding and reactor physics.

The first collision neutron dose (or “kerma,” see ref. [5]) at 0° with respect to the incident deuteron beam was measured as a function of distance in water from the D–D neutron source which emitted neutrons of 4.0-Mev maximum energy. This source is well-defined in energy versus angle. It should be possible to make a critical check between this experiment and appropriate penetration calculations. Unfortunately, at present there are no calculations which can be compared directly to our experiment hut Goldstein [6] has calculated the dose expected for a 2- and a 4-Mev monoenergetic, isotropic source. It would be expected that a similar calculation made using our D–D spectrum would show the dose to be between the 2- and the 4-Mev curve with its slope approaching that of the 4-Mev curve at large distance.

The absolute neutron dose was not measured in this experiment because it was not possible to measure accurately the beam current or the pressure of the gas target. The associated particle method of obtaining an absolute measurement was undesirable due to the duct effect of the associated equipment. Consequently, only the slope of the experimental curve can be compared to the calculations of Gold- stein.

## 2. Experimental Arrangement

**Figure 1:**
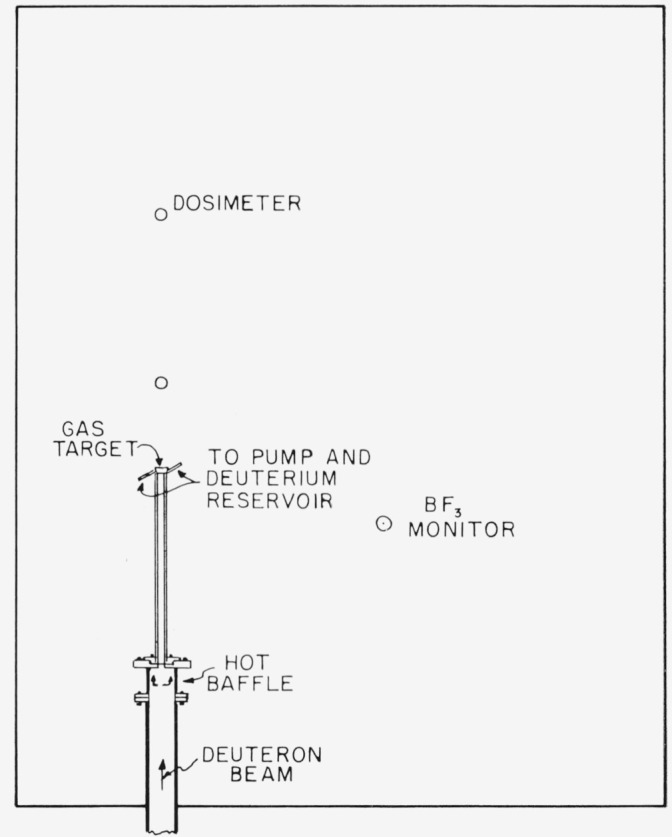
Experimental arrangement.

Neutrons were produced by the D(*d*,*n*)He^3^ reaction using an analyzed deuteron beam from a 2-Mev Van de Graaff accelerator. The deuteron beam impinged upon a modified gas target of the type described by Richardson [7] which was located in a tank of water 115 cm wide × 142 cm long × 60 cm deep. The gas cell of the target was coupled to the 2-in. beam tube by a 2 cm diam by 30 cm long thin wall brass tube in order to reduce duct effects. The gas cell was positioned 26 cm from the nearest wall and 30 cm from the bottom and 28 cm from the top of the tank. This arrangement is shown in figure 1.

The gas target had a cell 1.24 cm long and used a 0.1 mil nickel entrance foil. The entrance aperture was 38-in. diam and the impinging deuteron beam was first collimated by a heated baffle so that the remaining beam would completely pass through the entrance foil into the gas cell. The heated baffle is used to collimate a deuteron beam without production of undesired neutrons by the D(*d*,*n*)He^3^ reaction. The foil was cooled by continuously circulating the deuterium gas. This permitted the use of 2-*μ*a current for as long as 30 hr without burning out the foil. The pressure was maintained at 760 mm Hg in the cell. This target differed from the one described by Richardson in that a thick gold backing was used instead of an exit foil. The gold backing was rotated frequently in order to reduce the buildup of a “drive-in” target.

**Figure 2:**
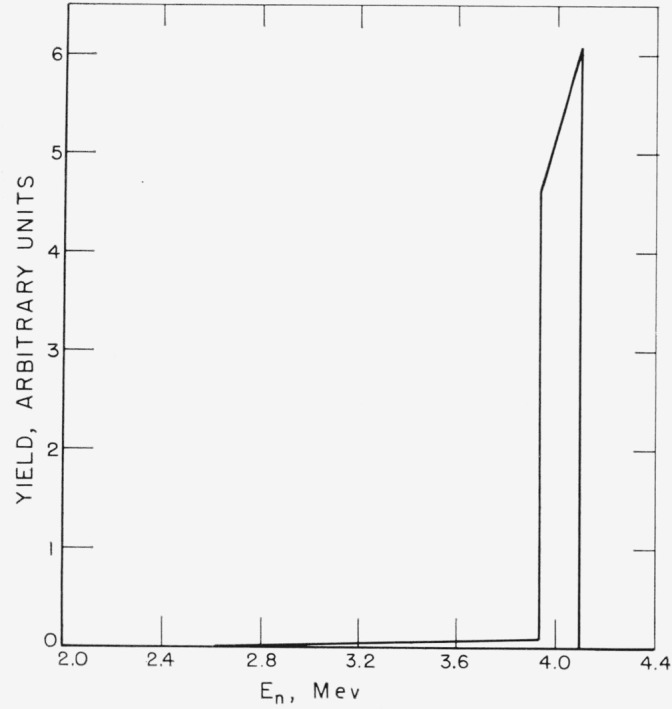
Neutron spectrum at 0° (gas target plus gold backing).

**Figure 3:**
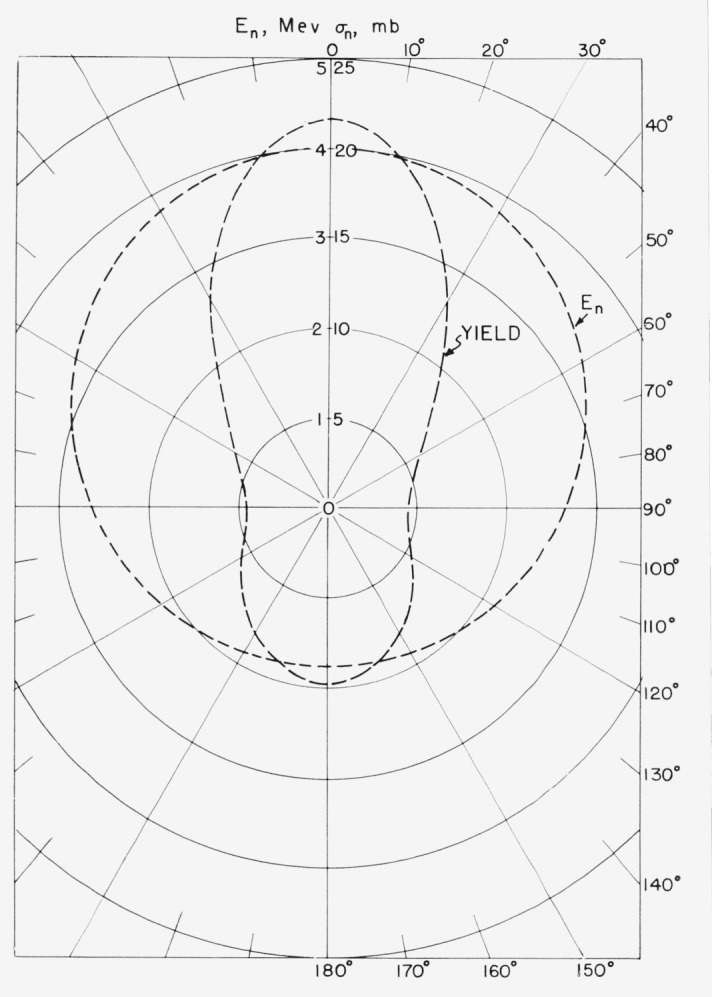
Angular and energy distribution of the D–D source. The differential cross section (in mb/ster) is shown by the dotted curve marked yield. The average energy at any given angle is given by the intersection of the off center dotted circle marked *E_n_* with a radius vector drawn from the origin in that direction. The concentric circles give the scale for both yield (mb/ster) and energy (Mev).

The deuteron beam incident energy was 1.36-Mev. Using the data of Whaling [8], the average energy loss of the beam in passing through the 0.1 mil entrance foil was computed to be 380-kev and that in the gas cell to be 150-kev. The resultant average deuteron energy in the gas cell was 0.905-Mev which produced neutrons of 4.0-Mev average energy at 0° with a neutron energy spread of 180-kev. A small number of lower energy neutrons were also produced by the formation of a “drive-in” target in the gold backing of the gas cell. Their relative intensity was measured by first forming a “drive-in” target by bombarding the gas cell with a deuteron beam for approximately 5 hr. The yield at 0° in air of the deuterium-gas filling was then measured and compared to the yield obtained by replacing the deuterium with hydrogen. This measurement showed that 3.1 percent of the neutron flux at 0° was due to “drive-in” neutrons. The idealized neutron spectrum from both the gas target and the gold backing is shown in figure 2. The angular distribution of the neutron yield [9] and energy [10] are shown in figure 3.

**Figure 4:**
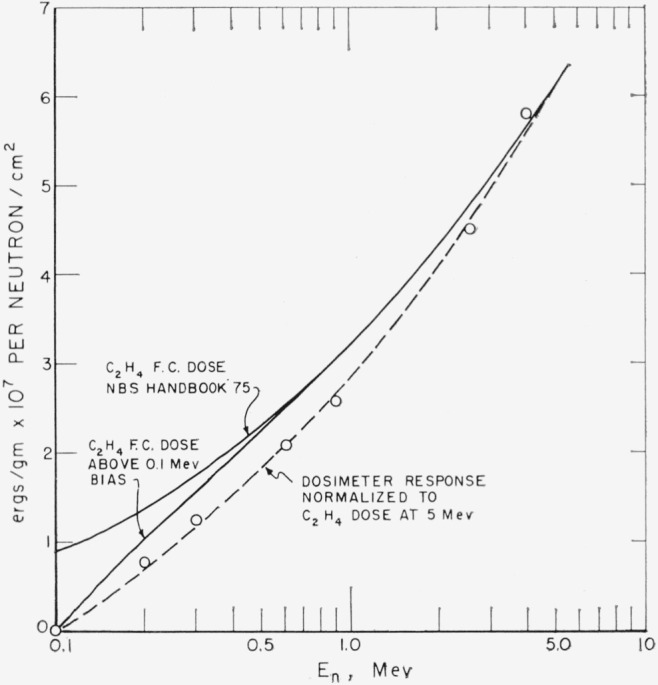
Dosimeter response.

A polyethylene-ethylene proportional counter dosimeter modeled after the secondary counter described by Hurst [11] was used to measure the neutron first collision dose [12]. This counter and its energy response is described in detail by Caswell et al. [13]. The energy response is shown in figure 4.

The signal was brought out of the dosimeter by a 3-ft RG–59/U coaxial cable passed through a 38 in. thin-wall, watertight brass tube attached to one end of the dosimeter. The other end of the brass tube was attached to and passed through a small (4 in. × 2 in. × 38 in.) brass plate which had two parallel grooves shaped to slide along a track. This track was mounted horizontally at the top of the water tank just above the water level and parallel to the beam direction. This permitted the dosimeter to be dropped into the water and positioned at various distances along 0° with its long axis vertical and perpendicular to the beam direction. The dosimeter was operated at 2150 v. High voltage was supplied by a regulated power supply.

The signal was bought from the dosimeter to a gain-of-sixteen, transistorized preamplifier powered by batteries. The signal was then carried by a 40-ft cable through a variable attenuator to an RCL linear amplifier and 256-channel analyzer. A constant pulse from a mercury-relay pulser could be fed into the input of the preamplifier thus permitting a quick check on the overall gain of the electronics and the gain could be quickly adjusted to the desired level by means of the variable attenuator.

It was necessary in the computing of neutron doses to reject small pulses which were caused by electrons ejected by gamma rays. Previous experience with this dosimeter [1] had indicated that the gamma ray contribution would be negligible if a 0.1-Mev bias were selected and all pulses falling below this bias were discarded. This was corroborated experimentally by first determining the 0.1-Mev bias by extrapolating the steep part of a 0.1-Mev T(P,*n*)He^3^ neutron spectrum to zero and then adjusting electronic gains so that this intercept would fall in channel 17. The dosimeter was then exposed to the 87-kev gamma ray of Cd^109^ and to the higher energy gamma rays of radium. All the pulses from the 87-kev gamma ray fell below the 0.1-Mev bias but a few pulses from the radium source fell as high as six channels above the bias. The dose falling above the 0.1-Mev bias was measured for the 4.0-Mev monoenergetic neutron source and for the radium source and these were compared when the total energy deposition in the dosimeter was the same for each. This neutrongamma ray discrimination ratio was found to be about 120. The 0.1-Mev cutoff as determined experimentally was reproducible to within one channel although the absolute accuracy was estimated to be no better than two or three channels.

The dosimeter was checked for possible drift by periodically measuring the dose in air from a plutonium-beryllium source. A slight drift was noticed when the energy calibrations were finished and the dosimeter was first placed in the water. The data were corrected for this drift and the resultant error due to dosimeter drift is estimated to be not greater than 5 percent.

## 3. First-Collision Dose Measurement

**Figure 5:**
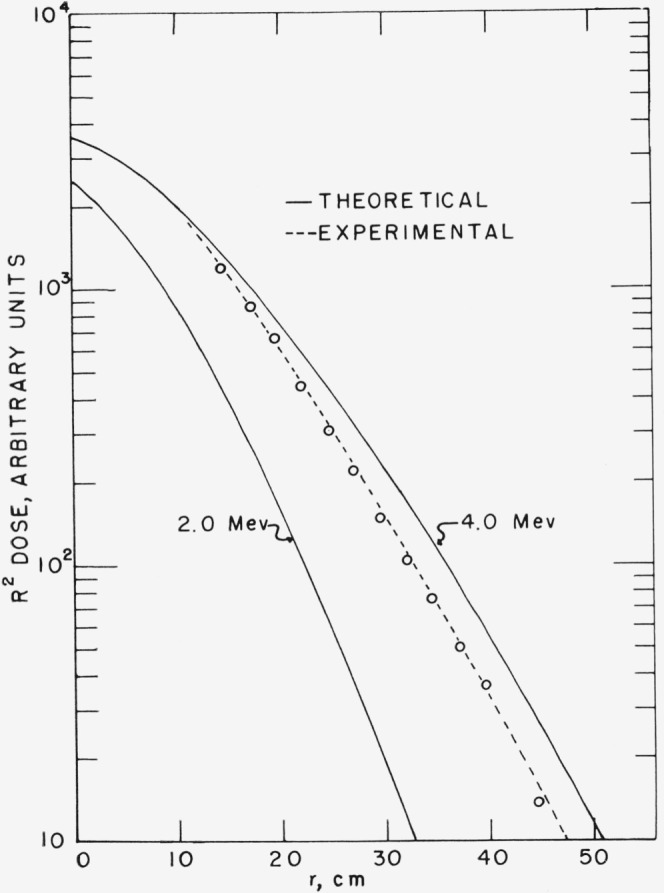
D–D neutron dose in water.

The D–D neutron first collison dose was measured as described by promenading the dosimeter at 2.5 cm intervals from 15 to 45 cm at 0° and then calculating the dose using a 0.1-Mev bias. The neutron flux was monitored during t hese runs by a cylindrical BF_3_ counter placed at 100° to the incident deuteron beam and approximately 40 cm from the target. The measured dose is given in table 1 and is plotted in figure 5. A computation of the dose was made using the neutron spectra of Goldstein [6] for a 2.0- and a 4.0-Mev neutron source and our dosimeter response and these are also shown in figure 5 with the experimental curve arbitrarily normalized to the 4.0-Mev curve at 10 cm. *R*, as given in figure 5 and table 1, is the distance from the center of the gas target to the center of the dosimeter, while *r* is the distance from the target-water boundary to the center of the dosimeter.

There was some uncertainty in determining the best value of *R* and *r* due to (1) the finite size of the neutron source and the variation in yield along its length, (2) the finite size of the dosimeter, and (3) the anisotropy of the neutron flux incident upon the dosimeter. In the ideal situation of a point source of neutrons located at the center of a void sphere of radius *r*_1_ and at a distance *r*_2_ from a point dosimeter, *R* would be given exactly by *r*_2_ while *r* would be given by *r*_2_—*r*_1_. In our experiment, the upper limit of the difference between the nominal values of *R* and *r* chosen above and the values of $\sqrt{\bar{R^{2}}}$ and $\sqrt{\bar{r^{2}}}$ computed for the worst case by taking into account the above effects was found to be small enough to warrant being ignored. The first effect would cause $\sqrt{\bar{R^{2}}}$ to be less than 0.02 cm longer than *R* while the second and third effects opposed each other and would cause $\sqrt{\bar{r^{2}}}$ to be less than 0.10 cm shorter than *r*.

The statistical errors for the first collision dose were computed by approximating the dosimeter spectrum with one of the form *Y=MX+B* where *Y* is the counts per channel and *X* is the channel. *M* and *B* are adjusted so that *Y*=0 for *X*=256 and $\int_{X_{I}}^{X_{F}}YdX=\text{True}\;\text{Counts}$. The standard deviation as computed in this manner will be given by

$$\delta_{\text{Dose}}^{2}=\frac{X_{F}^{4}}{6{\left(X_{F}-X_{I}\right)}^{2}}\sum_{i=I}^{F}Y_{i}$$

where *X_I_*=channel 18, *X_f_*=channel 256, and $\sum_{i=1}^{F}Y_{i}=\text{total}\;\text{counts}$ above the 0.1-Mev bias. The standard deviation as computed in this manner will be larger than the true standard deviation. When two or more commensurate runs were made at the same distance, they were averaged together by weighting each measurement according to its separate standard deviation.

## 4. Conclusion

A calculation to compare directly to this experiment is not yet available. However the 4- and 2-Mev monoenergetic, isotropic calculation of Gold stein [6] should bracket the experimental results. The slope of the penetration curve of this experiment is steeper at small distances but appears to approach at larger distances the slope of the 4.0-Mev calculation. This steeper slope close to the source is probably due to the contributions of neutrons of energies around 3.5-Mev which are emitted into forward angles, and which have a short mean free path and therefore scatter quickly and contribute to the dose seen by the dosimeter at 0°.

It is desirable that a calculation be made of the dose distribution at 0° with exactly the D–D neutron spectrum and angular distribution and the experimentally observed dosimeter response [13] of figure 4 to provide a more exact comparison with this experiment. Another possibility in the future is to extend the 0° curve to larger distances. This would require much higher currents on the gas target and greatly increased detector sensitivity.

## Figures and Tables

**Table 1:** D–D neutron first collision dose in water

First run at 0°		
*r*	*R*	R^2^ dose, arbitrary units
		
cm	cm	
14.25	14.87	1202.5±7.4
16.95	17.57	865.3±5.9
19.27	19.89	667.0±8.0
21.84	22.46	442.4±5.5
24.41	25.03	318.0±3.5
26.79	27.41	221.7±3.1
29.42	30.04	149.5±2.4
32.03	32.65	105.2±1.6
34.36	34.98	76.1±3.2
37.04	37.66	50.4±0.9
39.39	40.01	36.6±1.4
44.51	45.13	13.8±0.4

## Footnotes

1 Figures in brackets indicate the literature references at the end of this paper.
